# Author’s Response to Peer Reviews of “Interpreting the Estimand Framework From a Causal Inference Perspective”

**DOI:** 10.2196/98121

**Published:** 2026-05-22

**Authors:** Jinghong Zeng

**Affiliations:** 1 Department of Statistics, University of Auckland, 38 Princes Street, Auckland, 1010, New Zealand, 86 15992428924; 2 Department of Statistics and Programming, Jiangsu Hengrui Medicine (China), Guangzhou, Guangdong, China

**Keywords:** causal inference, clinical trial, estimand, intercurrent event, treatment effect

*This is the author’s response to peer-review reports for “Interpreting the Estimand Framework From a Causal Inference Perspective [1].”*

## Round 1 Review

### Reviewer G [2]

#### Major Comments

1. *The professional society “ICH” is never spelled out or introduced. Additional context is needed regarding the role of the ICH, its influence on regulatory science, and why its guidelines are particularly important for clinical trial design and analysis.*

**Response:** I’ve added an introduction to the International Council for Harmonisation of Technical Requirements for Pharmaceuticals for Human Use (ICH) to the first paragraph of the Introduction section.

2. *The Efficacy Guideline E9 was published in 2019. The authors should clarify what impact this guideline has had on the pharmaceutical industry since its release.*

**Response:** I’ve added an introduction to E9 and E9 (R1) to the first paragraph of the Introduction section.

2. *Moreover, it is unclear why a causal interpretation of this guideline is timely and important in 2025, several years after its publication.*

**Response:** I added some justifications for this aim in the second-, third-, and fourth-to-last paragraphs. The estimand is different from a causal treatment effect, so a causal interpretation could be helpful.

3. *None of the proposed strategies address noncompliance, such as cases where treatment is not received despite assignment or is received without assignment (eg, X(R=1)=0 or X(R=0)=1). Noncompliance is a central issue in causal inference and should be explicitly discussed. If noncompliance is assumed to be irrelevant, then the introduction of the notation R appears redundant and should be justified or removed.*

**Response:** Yes, noncompliance is an important issue in causal inference. Since causal inference is a broad area, this article is not intended as a review of causal inference. Only unmeasured confounding is discussed as an example. Noncompliance is not discussed further, but is mentioned in the Introduction section. The notation R is needed for discussion of unmeasured confounding and the intention-to-treat (ITT) principle.

4. *The strategies are presented at a very high level. Although the 4 cases illustrated in Figure 2 provide some intuition regarding the appropriateness of each strategy, the Viewpoint would be substantially strengthened by grounding the discussion in real clinical trial examples. Demonstrating how each strategy has been applied in practice would greatly improve clarity and impact.*

**Response:** I’ve added real clinical trial examples and explained them.

5. *The scope and framing of the Viewpoint appear better suited for a pharmaceutical science or regulatory-focused journal rather than a JMIR-based journal. The authors should better justify the relevance of this work to the JMIR readership or reconsider the target venue.*

**Response:** I think the paper suits the broad audience of this journal because interpretation from a causal inference perspective works not only for clinical trials in the pharmaceutical industry, but also for clinical trials in academic and observational studies. I’ve added a justification in the Introduction section.

#### Minor Comments

1. *Section 2 begins with the statement: “A causal inference framework is based on the potential outcome framework.” This is inaccurate, as causal inference can also be grounded in other frameworks, such as structural causal models.*

**Response:** Sorry for the inaccuracy. I only meant to say that the potential outcome framework is discussed. I’ve modified this sentence and added a clarification to the Introduction section.

2. *In the abstract, the sentence “This article aims to interpret the estimand framework through its underlying theories, the causal inference framework based on potential outcomes” should replace the comma with “and” for grammatical correctness.*

**Response:** I’ve revised the abstract and deleted this sentence.

3. *On page 2, second line: “Generally, Treaments are…” — the “T” in “Treatments” should not be capitalized.*

**Response:** I’ve removed the capitalization of this letter.

4. *In section 3.2 (page 6): the sentence “Through the hypothetical strategy, we make the second…” is ambiguous and should be rewritten for clarity.*

**Response:** I’ve rewritten the discussion about intercurrent event strategies. I’ve deleted this sentence.

### Reviewer H [3]

#### Major Comments

1. *The introduction may give the impression that these strategies are newly proposed by the author, whereas they are in fact defined in ICH E9 (R1). The manuscript would benefit from clearer attribution to, and positioning relative to, the ICH E9(R1) estimand framework.*

**Response:** I have clearly attributed the estimand framework to ICH E9 (R1) in the Introduction section.

2. *The important concept of intercurrent events is not clearly defined. The definition provided in the manuscript, “Intercurrent events are events that happen after treatment initiation and affect the definition of a treatment effect” (page 2) is vague and potentially misleading. It misses the key idea that intercurrent events are posttreatment events that interfere with the interpretation or existence of the outcome relative to the treatment of interest, rather than merely events that affect treatment effects.*

**Response:** I’ve improved the definition of intercurrent events according to the original definition in ICH E9 (R1) in the Introduction section.

3. *In section 2, it is incorrect to state that “Ri, Xi and Yi are potential outcomes.” Only Xi (⋅) and Yi (⋅) are potential outcomes. The randomization indicator Ri not a potential outcome; it is a realized random variable determined by the design.*

**Response:** I have not made changes. I disagree with this comment because the randomization scheme R here is also a potential variable. When we imagine a participant being randomized to a treatment arm or a control arm, we are imagining two different randomization outcomes. A participant cannot be assigned to two arms at the same time, so the two randomization outcomes are potential: they have not yet been realized when we consider potential outcomes of X and Y.

4. *At the beginning of section 2, the authors assume “an ideal two-arm randomized controlled clinical trial, with full compliance to treatment and no intercurrent events.” In such a setting, confounders do not affect treatment assignment. However, the manuscript later defines “some confounders C that affect both X and Y,” which contradicts the assumption of randomization.*

**Response:** Yes, I agree. To make the description more accurate, I have deleted the word “ideal.” I have also deleted “and no intercurrent events.”

5. *Average treatment effect* (*ATE) is defined as ATE = E(Y (X(R = 1) = 1) | C) − E(Y (X(R = 0) = 0) | C). However, this is a conditional ATE rather than the marginal ATE, since Y (X(R = 1) = 1) and Y (X(R = 0) = 0) are potential outcomes. The author should define the ATE marginally and then mention conditioning on C for adjustment.*

**Response:** Initially, I tried to write the methods in a simple mode to help readers understand, but this reduced some theoretical accuracy. I have fully updated the description of the causal inference framework with more rigorous research findings from my recently published paper. The ATE now is marginal for the whole article.

6. *Page 3: the phrase “the difference (D) between the average treatment effect from participants who take the experimental treatment...” is incorrect. The quantity described is the difference in average observed outcomes, not an average treatment effect.*

**Response:** I’ve updated the description of the causal inference framework. I deleted D.

7. *Across strategies, the author repeatedly claims that the estimand formula is “still” the same, which is misleading. The symbolic form may look similar, but the estimand is not the same. In treatment policy, X is redefined; in composite and while-on-treatment strategies, Y is redefined; in principal stratification, the target population changes. This undermines the central E9 (R1) message that different strategies define different estimands.*

**Response:** Sorry for the lack of clarity. I did not intend to undermine the estimand framework. In fact, I think highly of it. I have rewritten all descriptions of intercurrent event strategies.

8. *The proposed “model adjustment strategy” does not correspond to an estimand strategy as defined in ICH E9 (R1), but rather to a particular modeling or estimation approach. Moreover, in case 1 of Figure 2, concomitant therapies occur after treatment initiation, which is inconsistent with the causal diagram in Figure 3. In this setting, M may act as a mediator rather than a confounder. Treating postrandomization intercurrent events as confounders requires careful causal justification and may induce bias; this issue is not discussed in the manuscript.*

**Response:** Sorry for the oversimplification. After consideration, concomitant therapies after treatment initiation may indeed act as a mediator and also as a confounder for subsequent treatment. Indeed, this case can be complicated. After revision, I think the model adjustment strategy does not fit the article focus well, so I have deleted this section.

#### Minor Comments

9. *Please spell out the abbreviation “ICH” at its first occurrence.*

**Response:** I’ve spelled out ICH.

10. *Some sentences are confusing and would benefit from revision. For example, in the second paragraph on page 2: “Intercurrent events are frequent in practice but conceptually novel. E9(R1) listed many examples for intercurrent events, such as use of concomitant therapies, treatment switching and death before endpoint measurement.” Intercurrent events are not really new conceptually; rather, they were newly formalized or explicitly emphasized in E9 (R1). In “examples for intercurrent events,” the preposition should be “of,” not “for.” As a second example, “This individual treatment effect controls confounders on the endpoint within the same participant and means how the endpoint would change when only the treatment condition changes” on page 3: the ITE does not “control confounders”; it is defined counterfactually for the same individual.*

**Response:** I have revised “Intercurrent events are frequent in practice but conceptually novel. E9(R1) listed many examples for intercurrent events, such as use of concomitant therapies, treatment switching and death before endpoint measurement.” The new version is as follows: “Intercurrent events are very common in practice, including use of concomitant therapies, treatment switch and death before endpoint measurement.” Also, I have revised “This individual treatment effect controls confounders on the endpoint within the same participant and means how the endpoint would change when only the treatment condition changes” as follows: “The ITE means how the endpoint would change when only the treatment condition changes for this participant,” where ITE has been spelled out.

11. *A right parenthesis is missing in the first ATE formula on page 3.*

**Response:** Now the formula is complete.

12. *Equation 2.1 is missing the observed randomization indicator R^o in the first line.*

**Response:** I have deleted this equation, since I have updated the causal inference framework description.

13. *The exclusion restriction assumption for instrumental variables should be stated more clearly.*

**Response:** Many important assumptions are not mentioned in this article, as they are very technical. I have provided a reference to a complete list of assumptions instead.

### Reviewer P [4]

#### Major Comments

1. *The manuscript repeatedly states that it “interprets” the ICH E9 framework, but in practice, it mostly rephrases ICH E9 concepts using potential outcomes notation. Readers would more likely expect to see discussions on limitations, ambiguities, or contested aspects.*

**Response:** I have added more discussion of real clinical trial examples. I have also discussed differences between estimands and causal treatment effects.

2. *While pedagogical simplicity may be intentional, several aspects risk being misleading if read uncritically. For example, conditioning on posttreatment variables (section 3.6) is introduced without adequate warning about collider bias or causal ordering issues, and the discussion of principal stratification glosses over identification challenges, relying on brief mentions of Bayesian methods without clarifying assumptions. These are not fatal flaws, but the author should be more explicit about what is heuristic versus formally justified.*

**Response:** Yes, the discussion of conditioning on posttreatment variables requires more detail. Sorry for the oversimplification. However, this topic became less relevant to the revised paper, so I have now deleted this section. The article now focuses on a comparison between estimands and causal treatment effects, so the discussion of principal stratification now primarily serves this focus, and modeling approaches are not mentioned anymore.

## Abbreviations

ATE: average treatment effect
ICH: International Council for Harmonisation of Technical Requirements for Pharmaceuticals for Human Use
ITT: intention-to-treat
